# Supplementary material for: Responding to eruptive transitions during the 2020–2021 eruption of La Soufrière volcano, St. Vincent
Source: Nat Commun. 2022 Jul 15;13:4129. doi: 10.1038/s41467-022-31901-4 (PMC9287448; doi:10.1038/s41467-022-31901-4)
Supplement: Supplementary file 1 — Supplementary Information [file 41467_2022_31901_MOESM1_ESM.docx]

**Supplementary Information**

**
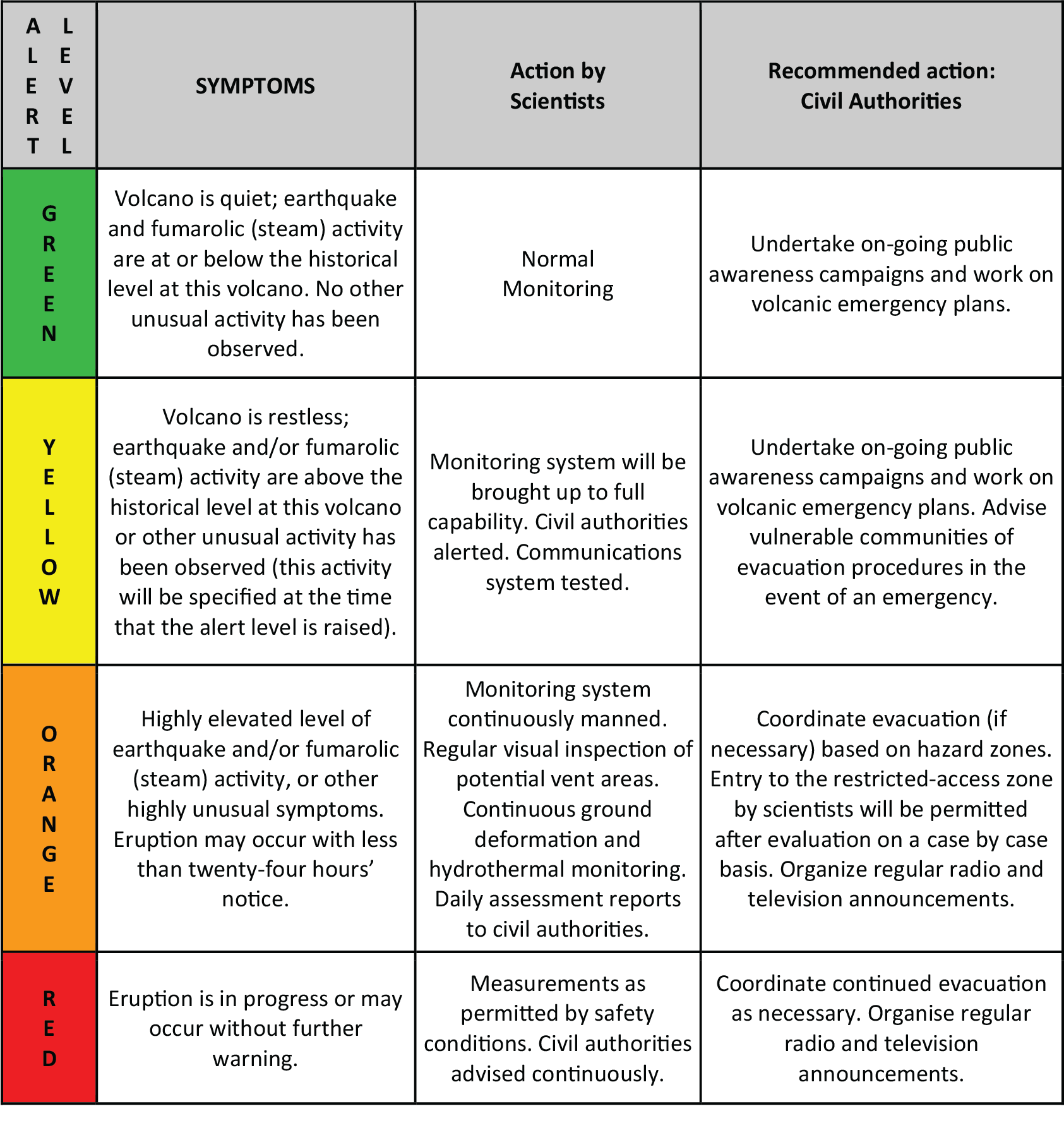
**

1. Volcanic Alert Level (VAL) Table for Onshore volcanoes in the Eastern Caribbean. At any given time, the alert level reflects the status of the volcano. Depending on the activity the alert level may change. These changes are determined by scientists at the UWI Seismic Research Centre in conjunction with the Government of St. Vincent and the Grenadines.


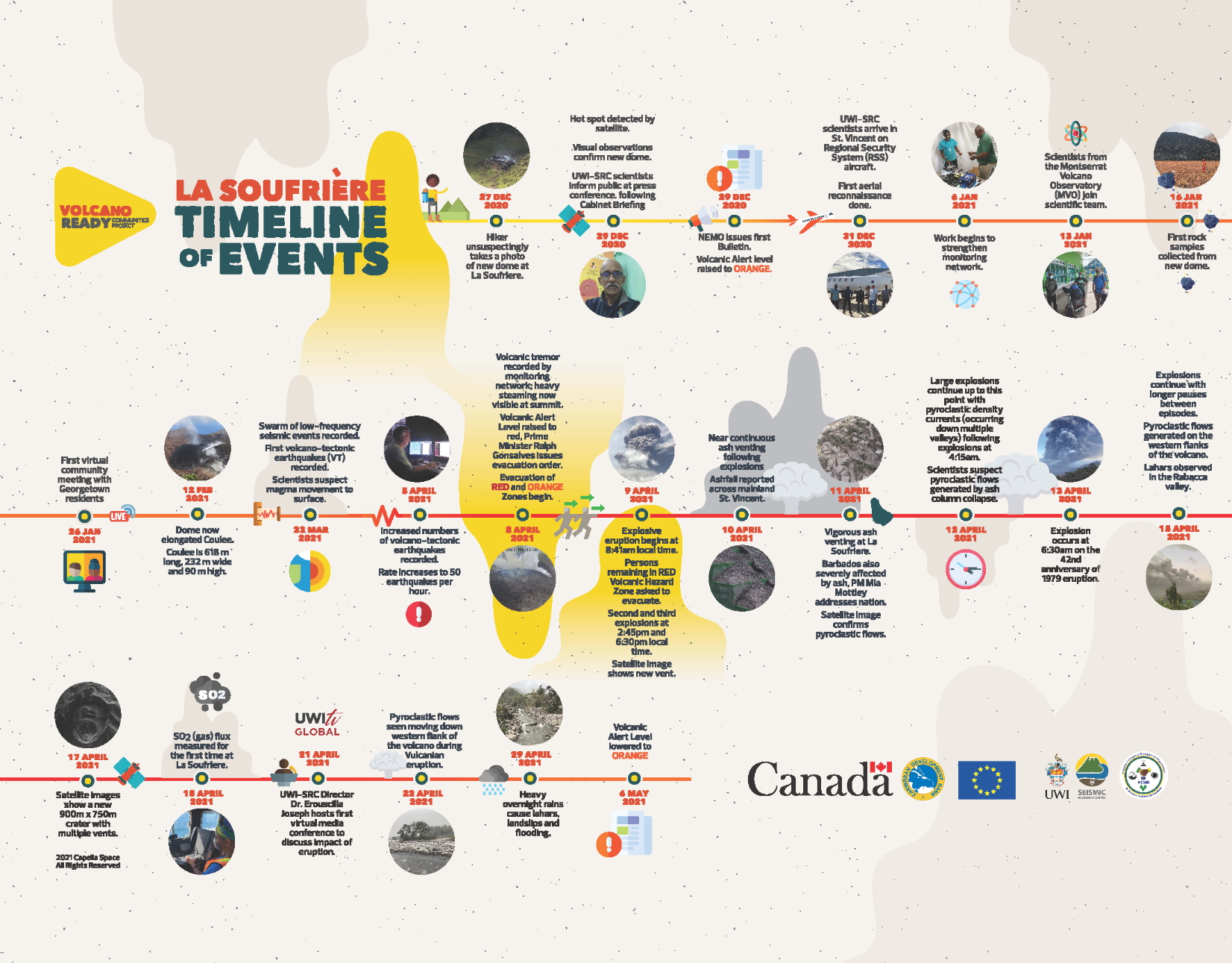


1. Timeline of the 2020-2021 eruption of La Soufrière volcano, St. Vincent from 27 December, 2020 to 6 May, 2021 (clipart sourced from https://www.flaticon.com).


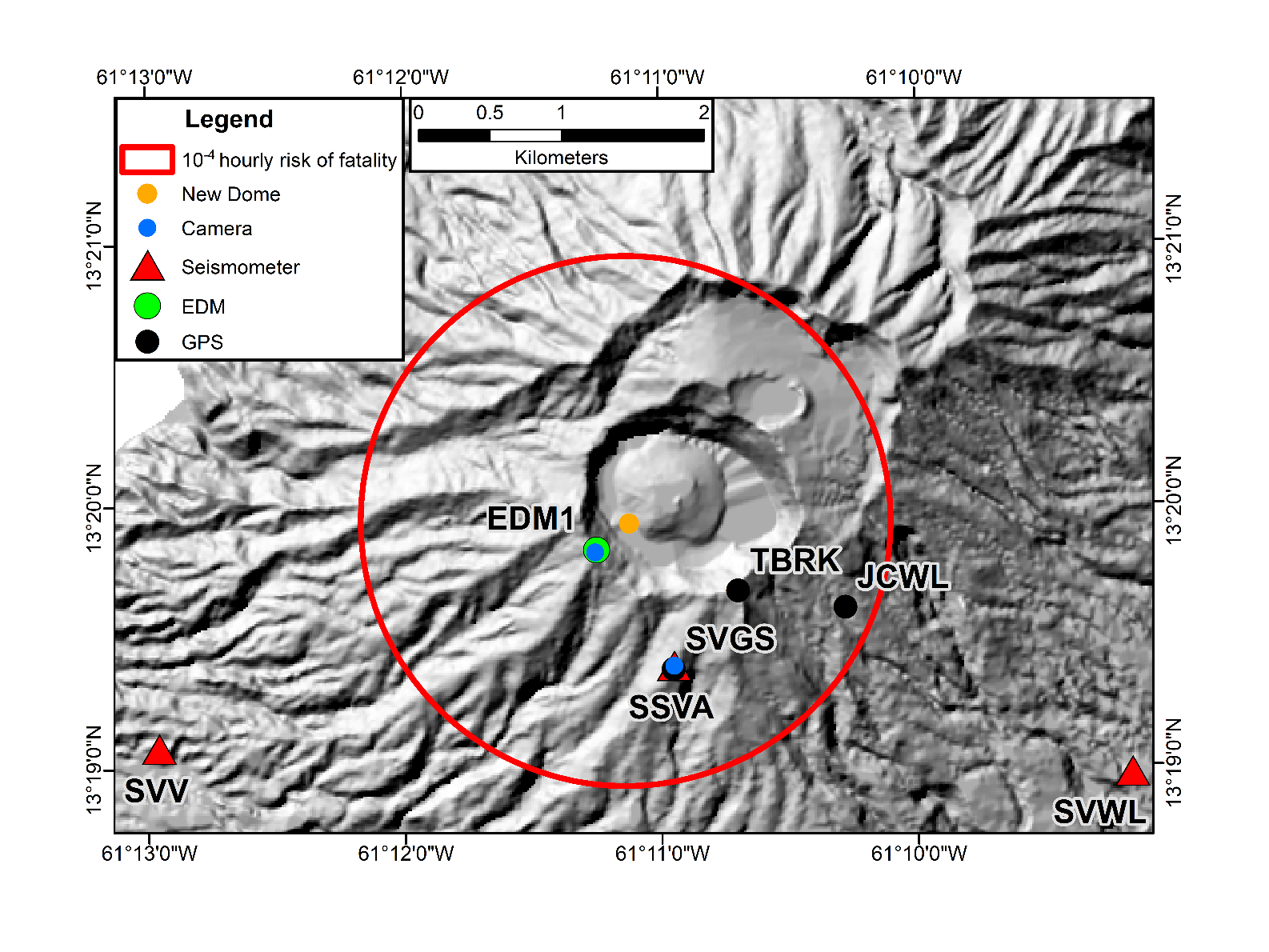


1. Map showing the estimated hourly risk of fatality exceeding a threshold of 10^-4^ in relation to monitoring stations established during the initial fieldwork period of network strengthening and sampling from the new dome on 16 January 2021. The fieldwork life-safety risk assessment was undertaken using the VoLREst methodology^41^ and eruption probabilities were determined through the expert-elicitation for anticipated eruption scenarios.
